# Learning to avoid looking: Competing influences of reward on overt attentional selection

**DOI:** 10.3758/s13423-020-01770-3

**Published:** 2020-06-30

**Authors:** Daniel Pearson, Mike E. Le Pelley

**Affiliations:** 1 Institute of Cognitive Neuroscience, University College London, 26 Bedford Way, London, WC1H 0AP England; 2 School of Psychology, UNSW Sydney, Sydney, NSW Australia

**Keywords:** Attentional capture, Reward, Suppression, Cognitive control

## Abstract

Pairing a stimulus with large reward increases the likelihood that it will capture attention and eye-gaze, even when such capture has negative consequences. This suggests that a stimulus’s *signalling relationship* with reward (the co-occurrence of that stimulus and reward) has a powerful influence on attentional selection. In the present study, we demonstrate that a stimulus’s *response relationship* with reward (the reward-related consequences of attending to that stimulus) can also exert an independent, competing influence on selection. Participants completed a visual search task in which they made a saccade to a target shape to earn reward. The colour of a distractor signalled the magnitude of reward available on each trial. For one group of participants, there was a negative response relationship between making a saccade to the distractor and reward delivery: looking at the distractor caused the reward to be cancelled. For a second group, there was no negative response relationship, but an equivalent distractor–reward signalling relationship was maintained via a yoking procedure. Participants from both groups were more likely to have their gaze captured by the distractor that signalled high reward versus low reward, demonstrating an influence of the signalling relationship on attention. However, participants who experienced a negative response relationship showed a reduced influence of signal value on capture, and specifically less capture by the high-reward distractor. These findings demonstrate that reward can have a multifaceted influence on attentional selection through different, learned stimulus-reward relationships, and thus that the relationship between reward and attention is more complex than previously thought.

## Introduction

Stimuli that signal large reward are more likely to capture attention and gaze than stimuli that signal lesser or no reward – a phenomenon named *value-modulated attentional capture* (VMAC; for reviews, see Anderson, 2016; Failing & Theeuwes, 2018; Le Pelley, Mitchell, Beesley, George, & Wills, 2016; Watson, Pearson, Wiers, & Le Pelley, 2019). VMAC has been demonstrated even when capture is counterproductive, in that it results in the omission of reward that would otherwise be delivered. For example, in a study by Le Pelley, Pearson, Griffiths, and Beesley (2015), participants made a rapid eye movement to a shape-defined target on each trial to earn reward. The colour of a colour-singleton distractor signalled the magnitude of reward that could be earned for a rapid eye movement to the target on that trial: one colour signalled high reward and another colour signalled low reward. However, if the participant looked at the coloured distractor *before* the target, the reward for the trial was cancelled. Even though looking at the high-reward-signalling distractor was counterproductive (as it triggered the omission of a relatively large reward), participants came to look at it more often than the low-reward-signalling distractor, thereby cancelling more high rewards. Subsequent research has indicated that this overt attentional bias to signals of reward is largely immune to top-down control, in that it persists when participants are explicitly instructed that looking at the reward-signalling distractor will result in reward omission (Kim & Anderson, 2019; Pearson, Donkin, Tran, Most, & Le Pelley, 2015), and under search conditions that allow physically salient distractors to be suppressed (Pearson, Watson, Cheng, & Le Pelley, 2020). However, the magnitude of VMAC has been shown to increase when cognitive control resources are depleted (Watson, Pearson, Chow, et al., 2019), which suggests a limited role for top-down control processes in reducing (but not preventing) the likelihood of capture by signals of reward. Together, these results suggest that reward-signalling stimuli receive special priority within the visual attention system, such that there is little a person can do to prevent themselves from attending to a reward signal, regardless of what the consequences might be.

**Fig. 1:**
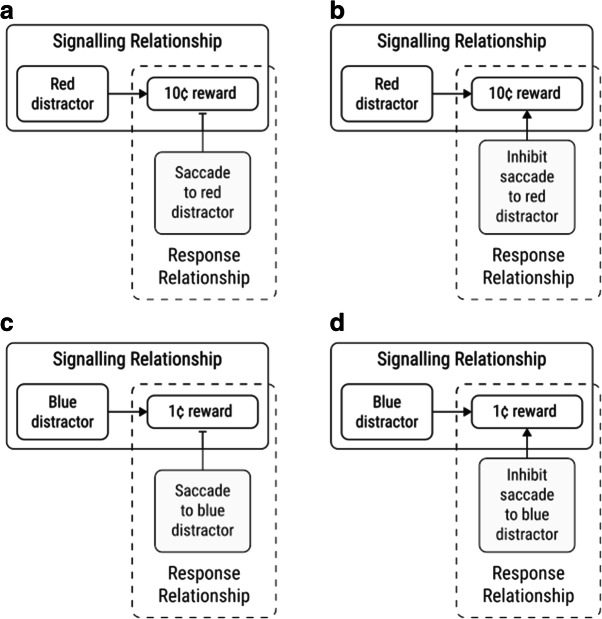
Diagrams of the signalling and response relationships for an example participant in Le Pelley et al.’s (2015) value-modulated attentional capture (VMAC) procedure. For this participant, red is the high-reward distractor colour and blue is the low-reward distractor colour. For each distractor type, there are two stimulus-reward relationships that the participant can learn. The signalling relationship (shown in the solid rectangle) refers to the statistical co-occurrence of the distractor and its associated reward. The response relationship (dashed rectangle) refers to the contingency wherein the reward will be omitted (indicated by the capped line) any time that the participant looks at the distractor (**a** and **c**). An alternative description of the response relationship is that the participant will receive the distractor's associated reward only when they successfully *inhibit* a saccade to it (**b** and **d**). As such, the motivation to inhibit saccades to the high-reward distractor should be higher than to the low-reward distractor. Consequently, if the response relationship has any effect on attention, it would be to reduce the extent to which the high-reward distractor captures gaze relative to the low-reward distractor: the opposite pattern to the standard VMAC effect

However, the true relationship between reward and attention may not be quite so simple. In many situations, the fact that a stimulus *signals* a rewarding outcome is only one of the stimulus-reward contingencies that are at play. For instance, in the previously described VMAC task (Le Pelley et al., 2015) there are two stimulus-reward relationships that participants could learn (see Fig. 1). Each distractor has a *signalling relationship* with its associated reward, i.e., a red distractor signals that the participant can earn 10¢ on that trial, and a blue distractor signals that they can earn 1¢. However, each distractor also has a *response relationship* with reward. This refers to the contingency between making a response (i.e., an eye movement^1^) to the distractor and the delivery of reward: for example, saccading to the red distractor results in omission of a potential 10¢; saccading to the blue distractor results in omission of a potential 1¢. To restate these response relationships, inhibiting a saccade to red (in this example) will result in a larger reward than inhibiting a saccade to blue. So, the motivation for participants to inhibit a saccade to red should be higher than that for blue. Thus, if a stimulus’s response relationship with reward influences attentional selection, its effect should be to encourage the opposite pattern of capture to the typical VMAC effect: *less* capture by the high-reward distractor than by the low-reward distractor.

Given the consistent empirical finding of *greater* capture by the high-reward distractor relative to the low-reward distractor, it must be the signalling pathway that is the primary driver of selection. One might assume that the response pathway therefore has no influence, such that the visual attention system completely disregards the consequences of attending to a stimulus when determining its attentional priority. Alternatively, the VMAC effect observed in behaviour may reflect the balance of the competing influences of the signalling relationship and the response relationship, with the signalling pathway tending to increase the attentional priority of the reward-related stimulus, and the response pathway tending to reduce it.

The current study investigated this possibility by testing whether a stimulus’s response relationship with reward influences attentional selection *independently* of its signalling relationship with reward. Put simply, are people better able to ignore a reward-signalling distractor when they have been repeatedly rewarded for ignoring it? There were two groups of participants. Each participant in group “Omission” experienced the standard version of the VMAC task used by Le Pelley et al. (2015), where the colour of a distractor signalled the magnitude of reward available for a rapid saccade to the target, and reward was omitted if the participant looked at the distractor. In a novel manipulation, each participant in group “Yoked” was matched to one participant in group Omission, such that they experienced the same sequence of trials and reward omissions as their matched pair, *regardless of whether or not they looked at the distractor*. Thus, the members of each matched pair experienced the same distractor-reward signalling relationships (each distractor co-occurred with its associated reward an approximately equal number of times) but different distractor-reward response relationships (looking at the distractor caused reward to be omitted for group Omission, but had no causal influence on reward delivery for group Yoked). That is, participants in group Omission were consistently rewarded for inhibiting saccades to the distractors whereas there was no consistent relationship between inhibiting saccades to the distractors and reward delivery in group Yoked. More specifically, participants in group Omission were particularly incentivised to inhibit saccades to the high-reward distractor, as doing so resulted in a larger reward than inhibiting saccades to the low-reward distractor. Thus, if a stimulus’s response relationship with reward influences attentional selection, participants in group Omission should exhibit a *smaller* VMAC effect relative to those in group Yoked. Moreover, this difference should specifically be driven by a reduction in capture by the high-reward distractor.

## Method

### Participants

Previous VMAC studies using gaze measures (Le Pelley et al., 2015; Pearson et al., 2015; Pearson et al., 2016) have demonstrated a mean effect size of *d*_*z =*_ 0*.*73 (range = 0.41–1.4). Based on an anticipated effect size of *d =* 0*.*73, a power analysis conducted with G*Power (Faul, Erdfelder, Lang, & Buchner, 2007) indicated that 48 participants per group would provide adequate power (~.95 for between-subjects *t*-tests, >.99 for within-subjects *t-*tests). We therefore tested for enough days to collect data from 96 participants (University of New South Wales (UNSW) Sydney students), with a final sample size of 98 (*n =* 49 per group, 62 females, age *M =* 20*.*4, *SD =* 3*.*85). Participants received a monetary bonus dependent upon their performance (*M =* 20*.*9 AUD, *SD =* 2*.*57 AUD).

### Apparatus

This study was approved by the UNSW Sydney Human Research Ethics Advisory Panel (Psychology). Participants were tested using a Tobii TX-300 eye-tracker (sampling frequency 300 Hz), mounted on a 23-in. monitor (1,920 × 1,080 resolution, 60 Hz refresh rate) with a chin rest ~60 cm from the screen. Gaze-contingent calculations used down-sampled gaze data (100 Hz). Stimulus presentation was controlled by MATLAB using Psychophysics Toolbox extensions (Brainard, 1997).

### Stimuli

**Fig. 2:**
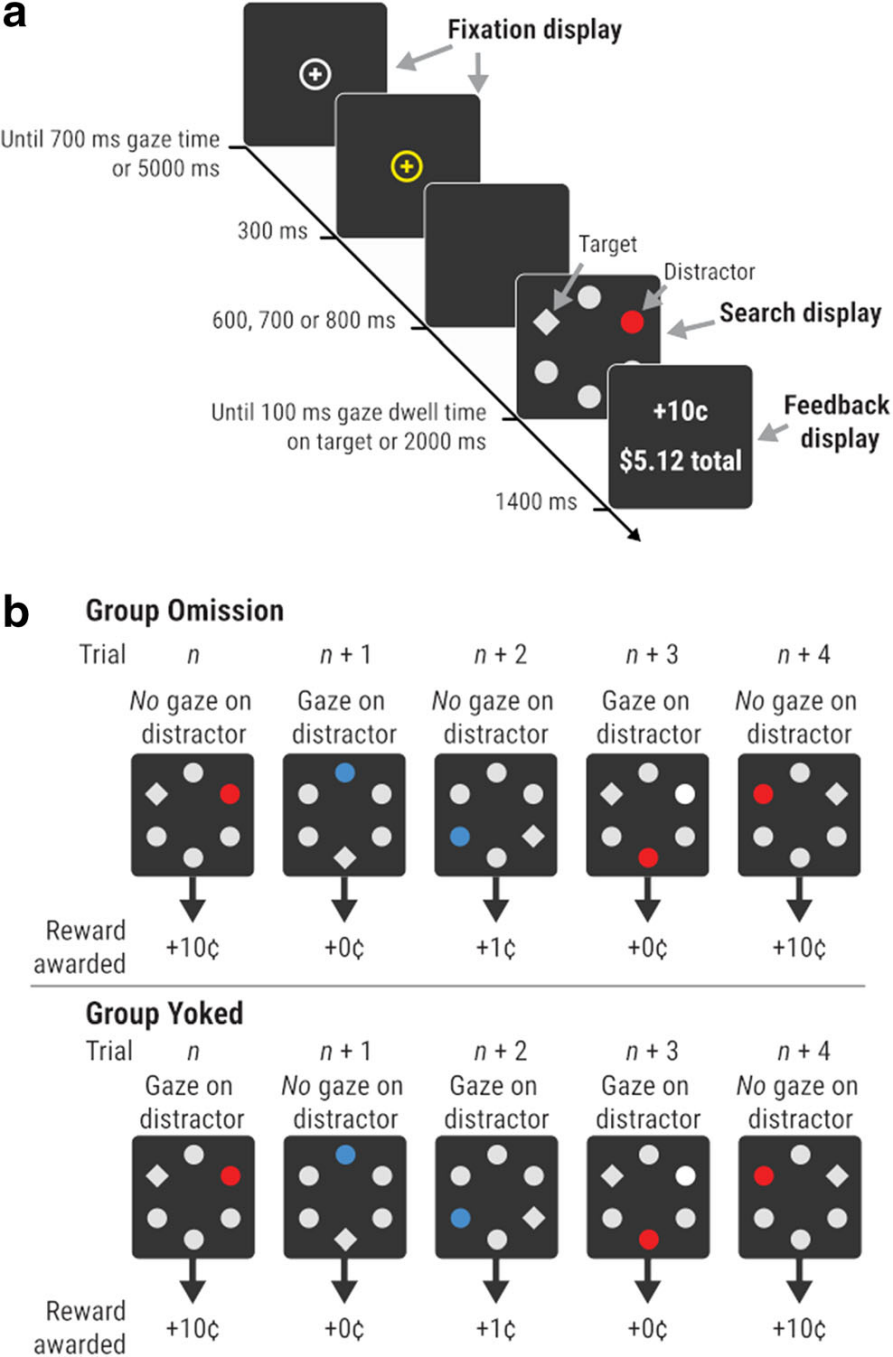
Design of the current task. (**a**) Participants began by fixating on a centrally presented fixation cross. A search display then appeared, and participants were required to make a saccade to a diamond-shaped target in order to earn reward. The colour of a colour-singleton distractor signalled the reward available on each trial (10¢ or 1¢). (**b**) Example sequence of trials for a participant in group Omission, and for the matched participant in group Yoked. For these participants, a red distractor signals availability of high reward (10¢) and a blue distractor signals low reward (1¢). For participants in group Omission, the sequence of trials and location of the target/distractor were randomly determined on every trial. In addition, reward was cancelled (i.e., an omission trial was triggered) if any gaze was detected on or near the distractor (trials *n* + 1 and *n* + 3 in the example shown here). For participants in group Yoked, the trial sequence and sequence of omission trials were yoked to a matched participant in group Omission

All stimuli were presented on a black background. Each trial consisted of a fixation display, search display and feedback display (see Fig. 2a). The fixation display consisted of a central white cross (0*.*5° × 0*.*5° visual angle) inside a white circle (diameter 3*.*0°). The search display comprised six filled shapes (2*.*3° × 2*.*3°) arranged evenly around the screen centre at 5*.*1° eccentricity (see Fig. 2). Five of the shapes were circles, and one (the *target*) was a diamond. The diamond target and four of the circles were grey (CIE *x*, *y* chromaticity coordinates: .304/.377, luminance ~32 cd/m^2^), with the remaining circle (the *distractor*) either red or blue (CIE coordinates: red .595/.360, blue .160/.116, luminance ~42.5 cd/m^2^), or the same shade of grey as the other shapes. The feedback display showed the reward for the previous trial, as well as the total reward accumulated.

### Design

Participants were alternately assigned to group *Omission* or group *Yoked*. For 25 of the participants in each group, red was the high-reward colour and blue was the low-reward colour; these relationships were reversed for the remaining participants. There were three types of trial: (1) *high-reward trials*, in which the distractor was rendered in the high-reward colour and a 10¢ reward was available; (2) *low-reward trials*, in which the distractor was rendered in the low-reward colour and a 1¢ reward was available; and (3) *distractor-absent trials*, in which all shapes were rendered in grey and there was an equal likelihood of 10¢ or 1¢ being available. The experiment comprised ten blocks of 48 trials. Each block consisted of 20 high-reward trials, 20 low-reward trials, and eight distractor-absent trials. For participants in group Omission, trial type was randomly determined within these constraints, and the location of the target and distractor were randomly determined with the constraint that the distractor was never presented adjacent to the target. For participants in group Yoked, trial type, target location, and distractor location on each trial were yoked to those of one participant in group Omission, such that each pair of matched participants experienced the *exact* same sequence of trials (i.e., the same sequence of trial types, with the target and distractor in the same positions across all trials; see Fig. 2b).

A circular region of interest (ROI) with a diameter of 3*.*5° was defined around the target, and a larger ROI (5*.*1°) was defined around the distractor. On distractor-absent trials, one of the non-target circles that was not adjacent to the target was randomly chosen to act as the ‘distractor’ location.^2^ A response was registered when 100 ms of gaze dwell-time was detected within the target ROI. Response times (RTs) slower than 600 ms were not rewarded. For group Omission, the reward for the trial was cancelled if any gaze was detected within the distractor ROI (hereafter called *omission trials*). By contrast, for group Yoked, omission trials were yoked to those of the participant’s matched pair (i.e., regardless of whether or not the Yoked participant looked at the distractor, a reward omission would be triggered on trial *n* if their matched pair from group Omission had looked at the distractor and triggered an omission trial on trial *n*; see Fig. 2b). Yoking the trial sequence and omission trials ensured that participants from each group had near-identical signalling relationships between each distractor and its associated reward (i.e., each distractor was paired with its associated reward equally often, disregarding timeouts), but different response relationships between each distractor and its associated reward (i.e., looking at the distractor resulted in the omission of reward for group Omission, whereas looking at the distractor had no direct effect on reward delivery for group Yoked).

### Procedure

Participants were told that their task was to move their eyes to the target as quickly and directly as possible on each trial, and that they could earn 0¢, 1¢ or 10¢ on each trial “depending on how fast and accurate” their response was, with responses slower than 600 ms receiving no reward.

Each trial began with the fixation display. Once 700 ms of gaze dwell-time was recorded within the circle surrounding the fixation cross, or after 5,000 ms, the cross and the circle turned yellow. After 300 ms the screen blanked, and after a random interval of 600, 700 or 800 ms the search display appeared and remained on screen until a response was recorded, or until 2,000 ms had passed. The feedback display then appeared for 1,400 ms. The inter-trial interval was 1,400 ms. Participants took a short break after every second block.

### Data analysis

In line with previous protocols (Le Pelley et al., 2015; Pearson et al., 2015), the first two trials of the task and the first two trials after each break were discarded. Hard timeouts (1.3% of all trials) were also discarded. Valid gaze data were registered on an average of 96.6% (*SD =* 5*.*9%) of samples.

Statistical analyses were conducted in R (Version 3.5.1). Greenhouse-Geisser corrected degrees of freedom are reported where appropriate. When conclusions are drawn on the basis of a null effect, we report the Bayes factor that corresponds to a Bayesian *t*-test using the default Cauchy prior.

Due to experimenter error, raw gaze coordinates were not saved during the experiment. However, data files containing aggregated gaze measures (i.e., a trial-by-trial recording of whether or not gaze was detected on the distractor, as well as gaze dwell-times on each distractor location) are available at https://osf.io/dy5kj/.

## Results

**Fig. 3:**
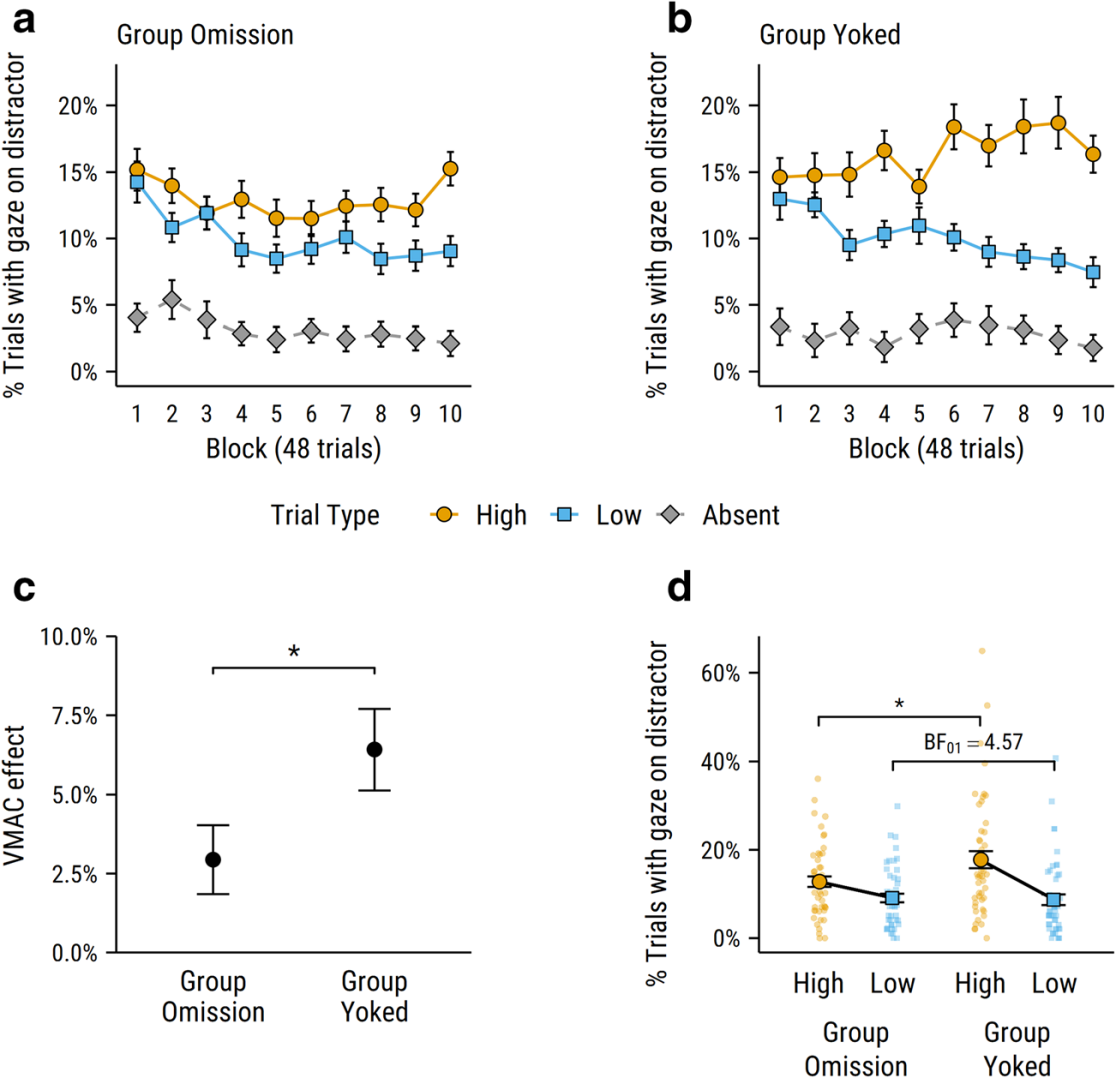
(**a** and **b**) Mean percentage of trials with gaze on the distractor across blocks for group Omission and group Yoked, respectively. (**c**) Mean VMAC effect, calculated as percentage of trials with gaze on high-reward distractor minus percentage of trials with gaze on the low-reward distractor, averaged across blocks. (**d**) Mean percentage of trials with gaze on each distractor in the second half of the experiment. Individual participant performance shown by faint, underlying points. Error bars in **a** and **b** represent within-subjects *SEM* (Morey, 2008). Error bars in **c** and **d** represent between-subjects *SEM.* *p < .05

Figure 3a and b shows the percentage of each trial type with gaze on the distractor (or the designated ‘distractor’ location on distractor-absent trials) over the course of the task for group Omission and group Yoked. Paired *t*-tests averaging across blocks confirmed that participants from both groups were more likely to look at the distractor location on trials containing a colour-singleton distractor than on distractor-absent trials: for group Omission – high versus absent, *t*(48) = 9*.*18, *p < .*001, *d*_*z*_ = 1.31; low versus absent, *t*(48) = 7*.*77, *p < .*001, *d*_*z*_ = 1.11; and for group Yoked – high versus absent, *t*(48) = 8*.*24, *p < .*001, *d*_*z*_ = 1.18; low versus absent, *t*(48) = 6*.*44, *p < .*001, *d*_*z*_ = 0*.*92.

Of more interest is the comparison between trials with a high-reward distractor versus a low-reward distractor, since this provides our measure of the effect of reward on attention. These data were analysed with a 2 (group: Omission, Yoked) × 2 (trial type: high-reward, low-reward) × 10 (block) ANOVA. This revealed a significant main effect of trial type, *F*(1*,*96) = 30*.*57, *p < .*001, η^2^_*p*_ = *.*242, with gaze more often directed to the distractor on high-reward trials than low-reward trials, demonstrating a VMAC effect overall. There was also a significant trial type × block interaction, *F*(7*.*56*,*725*.*44) = 3*.*74, *p < .*001, η^2^_*p*_ = *.*037, with the magnitude of the VMAC effect increasing linearly over the course of training, *t*(96) = 4.20, *p < .*001. Critically, there was also a significant group × trial type interaction, *F*(1*,*96) = 4*.*19, *p = .*043, η^2^_*p*_ = .042, with participants in group Omission showing a smaller VMAC effect than those in group Yoked (see Fig. 3c). Given that participants from each group experienced an identical sequence of trials and rewards, the implication is that group Omission’s experience of the response contingency between making a saccade to the distractor and the omission of reward resulted in a reduced VMAC effect relative to group Yoked, who experienced no such response contingency.

Follow-up *t*-tests were used to investigate the group × trial type interaction further. Paired-samples tests (high-reward vs. low-reward) confirmed the presence of a significant VMAC effect for group Omission, *t*(48) = 2*.*69, *p = .*010, *d*_*z*_ = 0.38, and for group Yoked, *t*(48) = 4*.*99, *p < .*001, *d*_*z*_ = 0.71. Orthogonal between-subjects tests revealed that, on low-reward trials, there was no significant difference in the percentage of trials with gaze on the distractor between group Omission (*M =* 10*.*0%, *SEM =* 0*.*9%) and group Yoked (*M =* 10*.*0%, *SEM =* 1*.*2%), *t*(96) = 0*.*03, *p = .*976, *d*_*s*_ = 0.01. For high-reward trials, while there was a trend towards participants in group Yoked (*M =* 16*.*4%, *SEM =* 1*.*6%) being more likely to look at the distractor than participants in group Omission (*M =* 12*.*9%, *SEM =* 1*.*2%), this contrast did not reach conventional significance levels, *t*(96) = 1*.*70, *p = .*092, *d*_*s*_ = 0.34.

The significant trial type × block interaction in the omnibus ANOVA reported earlier indicates that the VMAC effect increased over the course of training, as participants gained more experience with the colour-reward contingencies. The analyses of simple effects noted in the previous paragraph collapse across all trials: as such they include data from trials early in training, when participants have had relatively little experience with the colour-reward contingencies, so reward-driven differences in attention would be weak or non-existent. If analysis is restricted to the latter half of training (i.e., blocks six to ten), after participants have had extensive experience of the colour-reward contingencies (see Fig. 3d), then participants in group Omission were significantly less likely to look at the distractor on high-reward trials than participants in group Yoked, *t*(96) = 2*.*20, *p = .*030, *d*_*s*_ = 0.44. By contrast, there was no difference between the two groups in the rate of looking at the distractor on low-reward trials, *t*(96) = 0*.*25, *p = .*799, *d*_*s*_ = 0.05, with a corresponding Bayes factor of BF_01_ = 4.57, indicating moderate support for the null hypothesis. The implication is that experiencing the response relationship between looking at each distractor and the omission of the associated reward (in group Omission) specifically resulted in less capture by the high-reward distractor, but did not affect capture by the low-reward distractor.

## Discussion

Previous findings (e.g., Le Pelley et al., 2015; Pearson et al., 2015) have demonstrated that a stimulus’s signalling relationship with reward influences its likelihood of being selected by the visual attention system, such that stimuli that signal high reward are more likely to capture attention and gaze than stimuli that signal low reward. The experiment reported here goes beyond these prior studies by investigating whether a stimulus’s *response relationship* with reward (i.e., the reward-related consequence of attending to the stimulus) also influences attentional selection. In line with previous findings, participants were more likely to look at a colour-singleton distractor that signalled high reward than an equivalent distractor that signalled low reward, even when looking at the distractor triggered the omission of reward (i.e., both groups demonstrated a VMAC effect). This is consistent with the idea that a stimulus’s signalling relationship with reward induces a powerful increase in its attentional priority (Failing, Nissens, Pearson, Le Pelley, & Theeuwes, 2015; Pearson et al., 2016). Critically, however, the VMAC effect was *smaller* for a group of participants who experienced this negative response relationship with reward than for a group that experienced no negative response relationship, such that making a saccade to the distractor had no consequence for the delivery of reward. Thus, the key novel contribution of this study is to demonstrate that a stimulus’s response relationship with reward influences attentional selection *independently* of its signalling relationship with reward.

Previous studies have demonstrated that participants will prepare strategic top-down control when made aware that accurate performance in a visual search task will be highly rewarded (e.g., Sawaki, Luck, & Raymond, 2015; for a review, see Failing & Theeuwes, 2018). One possible explanation for the smaller VMAC effect observed here is that the participants who experienced the negative response relationship between looking at the distractor and reward engaged a similar strategy: i.e., after learning that looking at the distractors resulted in the omission of reward, participants in group Omission devoted additional resources to attentional control before each trial. This account would anticipate that participants who experienced the response contingency would show less capture by *both* the high- and low-reward distractors (as participants had no way of knowing in advance whether a high-reward or low-reward distractor would be present in the display on each trial). However, participants in group Omission showed a specific reduction in their rate of looking at the high-reward distractor, but not the low-reward distractor. This suggests that a stimulus’s response relationship has a feature-specific, *value-modulated* influence on distractor suppression, such that participants will inhibit saccades to distractors for which capture will result in the omission of high-value reward more than to distractors for which capture will result in the omission of low-value reward. Thus, the VMAC effect observed here (and in previous studies where the distractor signals reward and looking at the distractor triggers reward omission; e.g., Le Pelley et al., 2015) reflects the balance of two opposing value-modulated processes: the signalling relationship with reward increases capture, and the response relationship with reward increases suppression.

While the current findings suggest that a stimulus’s response relationship with reward can reduce oculomotor capture by that stimulus, the mechanism by which this occurs remains unclear. There are thought to be two different sets of mechanisms that the visual attention system uses to suppress capture by salient-but-irrelevant distractors (Geng, 2014): *proactive suppression*, where the attentional priority of the salient-but-irrelevant stimulus feature is down-weighted before the stimulus is ever presented to the observer, such that it never captures attention or gaze; and *reactive suppression*, where covert attention is rapidly disengaged from a stimulus after initial capture, but before the initiation of a saccade to that stimulus. Either of these mechanisms may be underlying the reduced VMAC effect observed in group Omission. Future studies could aim to tease apart these alternative mechanisms, potentially by examining the time course (e.g., Gaspelin, Leonard, & Luck, 2017) and/or trajectory of the eye movements (e.g., Van der Stigchel, Meeter, & Theeuwes, 2006) to the distractor (see also Watson, Pearson, Theeuwes, Most, & Le Pelley, 2020).

Moreover, it is unclear to what extent the influence of the response relationship on attention is an automatic consequence of repeatedly experiencing the omission contingency (i.e., is a consequence of *selection history*; Awh, Belopolsky, & Theeuwes, 2012; Failing & Theeuwes, 2018), or alternatively is a result of volitional top-down attentional control processes. Several previous studies have investigated the role that top-down control plays in reducing capture by reward-related stimuli. Pearson et al. (2015) found that participants could not reduce capture by reward-signalling distractors in a VMAC task when given explicit instructions about the negative response relationship between looking at the distractor and omission of reward. This was taken to suggest that the VMAC effect was immune to top-down attentional control processes. However, an alternative explanation is that participants were *already* devoting maximal resources to preventing capture by the reward-signalling distractors (and the high-reward distractor in particular) as a consequence of experiencing the omission contingency on a trial-by-trial basis, such that they could not reduce capture any further when explicitly instructed about it. In line with this idea, capture by reward-signalling stimuli is magnified when participants’ cognitive resources are otherwise engaged by a verbal working-memory task (Watson, Pearson, Chow, et al., 2019), which suggests that top-down control processes can play a (limited) role in reducing the magnitude of the VMAC effect. Similar processes could be responsible for the between-group differences observed in the current study, with participants in group Omission devoting more top-down control resources to reducing capture by reward-related stimuli as a consequence of experiencing the negative response relationship between looking at the distractors and reward delivery. Future studies could aim to elucidate the cognitive mechanisms underlying the response pathway’s influence on attention and determine the ways in which this influence of reward is similar to and/or different from that of the signalling pathway.

Notably, while participants in group Omission showed a reduction in their rate of looking at the high-reward distractor, this difference reached conventional significance levels only in the latter half of the experiment. By contrast, the VMAC effect itself was significant in analyses that collapsed across all experimental trials, and inspection of Fig. 3a suggests that the difference in the rate of looking at the high- and low-reward distractors emerged relatively early in the experiment across both groups. This suggests that participants learned about the signalling relationships more quickly than the response relationships in this task. There are at least two reasons why this may have been the case. First, there is a difference in the rate of delivery of the most relevant outcome for each type of relationship: while participants can learn the signalling relationship between each distractor and its associated reward on the 80–90% of trials in which reward is delivered, participants can only learn about the response relationship on the remaining 10–20% of trials in which they look at the distractor and a reward-omission is triggered. Moreover, as the explicit outcome for making a saccade to either the high- or low-reward distractor is 0¢, the difference between the two response relationships is directly dependent on what has already been learned about the signalling relationship between each distractor and its respective reward. That is, a reward omission on a high-reward trial is more punishing to the participant than a low-reward omission only if they have *already learned* that a high-reward distractor signals the potential to win a larger reward. As such, differential learning about response relationships can only occur after there has been differential learning about signalling relationships.

To summarise, the current findings complicate current theories of reward-related attention, which suggest that reward acts solely by increasing the attentional priority of paired stimuli (e.g., Anderson, 2016). Rather, our findings suggest that reward has a complex and multifaceted effect on attention, with independent stimulus-reward contingencies having independent (and sometimes opposing) influences on attentional selection.

## Footnotes

1 Because this experiment (and the experiment reported in this paper) used a gaze-contingent oculomotor capture task, we discuss the response relationship only in terms of the reward-related consequences of making an eye movement to the distractor. However, participants could potentially learn similar relationships between shifts of covert attention to the distractor and (loss of) reward in other VMAC tasks that rely on covert attention (e.g., Le Pelley et al., 2015, Experiments 1 and 2): the ‘response’ in a response relationship may not necessarily involve an overt behaviour.

2 Allowing for omissions on distractor-absent trials is useful for two reasons: (1) it allows for a valid test of the effect of physical salience on oculomotor capture, by comparing the omission rate on trials featuring a salient distractor with the rate on distractor-absent trials; and (2) it allows us to estimate the proportion of trials in which a reward omission is triggered for reasons other than oculomotor capture (e.g., inaccurate recording of gaze position, random inaccurate eye movements) since these factors will be equal across trials with a salient distractor and distractor-absent trials.
